# Human Amniotic Epithelial Cell Transplantation is Safe and Well Tolerated in Patients with Compensated Cirrhosis: A First-in-Human Trial

**DOI:** 10.1093/stcltm/szae023

**Published:** 2024-06-10

**Authors:** Rebecca Lim, Alexander Hodge, Sherryne Warner, Gregory T Moore, Jeanne Correia, Mirja Krause, Hannah McDonald, Siow T Chan, Mihiri Goonetilleke, Stuart M Lyon, William Sievert

**Affiliations:** The Ritchie Centre, Hudson Institute of Medical Research, 27-31 Wright Street, Clayton, Melbourne 3168, Australia; Department of Obstetrics and Gynecology, Monash University, 246 Clayton Road, Clayton, Melbourne 3168, Australia; Department of Gastroenterology, Eastern Health, 5 Arnold Street, Box Hill, Melbourne 3128, Australia; School of Clinical Sciences, Monash University, 246 Clayton Road, Clayton, Melbourne 3168, Australia; The John Goldman Centre for Cellular Therapy, Hammersmith Hospital, Ducane Road, London W12 OHS, United Kingdom; School of Clinical Sciences, Monash University, 246 Clayton Road, Clayton, Melbourne 3168, Australia; The John Goldman Centre for Cellular Therapy, Hammersmith Hospital, Ducane Road, London W12 OHS, United Kingdom; The John Goldman Centre for Cellular Therapy, Hammersmith Hospital, Ducane Road, London W12 OHS, United Kingdom; The Ritchie Centre, Hudson Institute of Medical Research, 27-31 Wright Street, Clayton, Melbourne 3168, Australia; The Ritchie Centre, Hudson Institute of Medical Research, 27-31 Wright Street, Clayton, Melbourne 3168, Australia; Department of Obstetrics and Gynecology, Monash University, 246 Clayton Road, Clayton, Melbourne 3168, Australia; The Ritchie Centre, Hudson Institute of Medical Research, 27-31 Wright Street, Clayton, Melbourne 3168, Australia; Department of Gastroenterology, Monash Health, 246 Clayton Raod, Clayton, Melbourne 3168, Australia; The Ritchie Centre, Hudson Institute of Medical Research, 27-31 Wright Street, Clayton, Melbourne 3168, Australia; Department of Obstetrics and Gynecology, Monash University, 246 Clayton Road, Clayton, Melbourne 3168, Australia; The Ritchie Centre, Hudson Institute of Medical Research, 27-31 Wright Street, Clayton, Melbourne 3168, Australia; Diagnostic Imaging Department, Monash Health, 246 Clayton Road, Clayton, Melbourne 3168, Australia; School of Clinical Sciences, Monash University, 246 Clayton Road, Clayton, Melbourne 3168, Australia; The John Goldman Centre for Cellular Therapy, Hammersmith Hospital, Ducane Road, London W12 OHS, United Kingdom

**Keywords:** liver fibrosis, cirrhosis, stem cells, phase I clinical trial, human

## Abstract

Placenta-derived human amniotic epithelial cells (hAEC) exhibit anti-inflammatory and anti-fibrotic effects in cirrhosis models. We conducted a first-in-human phase I clinical trial to assess the safety and tolerability of hAEC in adults with compensated cirrhosis. We examined increasing and repeated doses of hAEC in 9 patients in 3 cohorts. Cohort 1 patients received 0.5 × 10^6^/kg hAEC in one IV infusion. Cohort 2 patients received 1 × 10^6^/kg hAEC in one IV infusion. The patients in cohort 3 received 1 × 10^6^/kg hAEC on days 0 and 28. Here, we report follow-up to post-infusion day 56 (D56), during which no serious adverse events occurred. Six patients experienced no study-related adverse events, while 3 patients reported mild (grade 1) headaches that were possibly infusion-related. A transient decrease in serum platelet count occurred in all patients, which returned to baseline screening values by day 5. FIB-4 values to assess fibrosis were significantly lower at D56. Although not statistically significant, serum AST levels and liver stiffness measurements at D56 were lower than those at baseline. The hepatic venous pressure gradient, a measure of portal hypertension, declined in 4 patients, did not change in 3 patients, and increased in 2 patients. In conclusion, intravenous infusion of allogeneic hAEC in patients with compensated cirrhosis at the doses used in this study was safe and well tolerated, with no difference observed between 1 and 2 doses. Decreased hepatic inflammation, liver stiffness, and portal hypertension support larger studies aimed at identifying patients who may benefit from this therapy.

Clinical Trial registration: The trial was prospectively entered on the Australian Clinical Trials Registry (ANZCTR12616000437460).

## Graphical Abstract

**Graphical Abstract:**
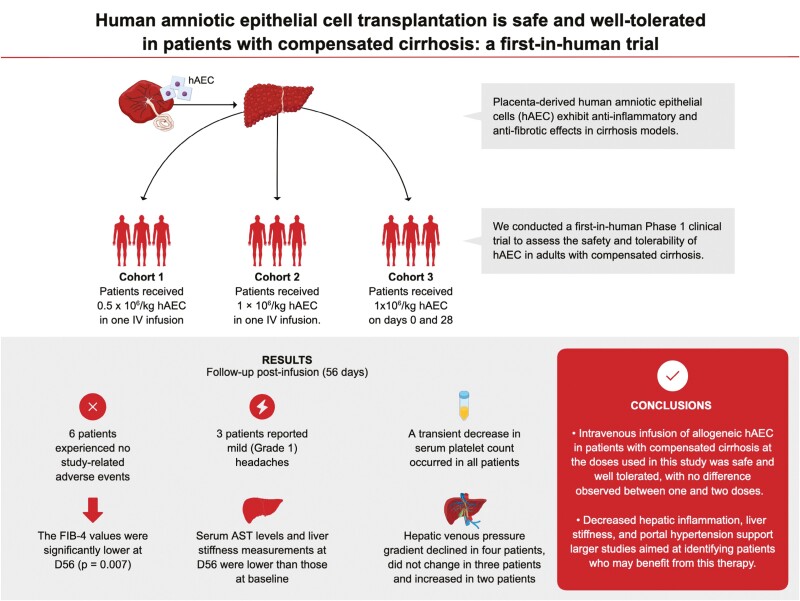

**Lessons Learned**

- Administration of stem cells derived from the placenta is safe and well tolerated in patients with cirrhosis with the doses used in this study.
- While this was a small study of 9 people with cirrhosis, we found that some of them had favorable changes in markers of liver inflammation and fibrosis.
- People with cirrhosis have different levels of liver injury (inflammation, fibrosis) so further studies are needed to understand which patients are likely to benefit from treatment with these placental stem cells.

**Significance Statement**

Liver transplantation is the only chance for survival in some patients with advanced liver disease but donor shortages mean that patients may die while waiting for a new liver. We showed in animal models that human amniotic epithelial cells (hAEC), derived from the placenta, reduce the severity of liver fibrosis and inflammation. We conducted a phase I, first-in-human clinical trial in 9 cirrhotic patients to determine whether giving hAEC in different doses was safe. No serious adverse events occurred during the trial and some patients showed reductions in liver inflammation and fibrosis markers. Our findings support additional clinical studies to identify which patients may benefit from this potential new treatment for cirrhosis.

## Introduction

The global burden of end-stage liver disease is high, with liver cirrhosis being the 11th, and liver cancer being the 16th most common cause of death. Together, these conditions account for 3.5% of the deaths worldwide.^1^ Cirrhosis is the outcome of a complex wound-healing response to persistent injury characterized by chronic inflammation, increased extracellular matrix production, and loss of hepatocyte mass. Although it was previously considered a static and irreversible condition, there is now evidence that liver fibrogenesis is dynamic and reversible. Robust clinical data show that cirrhosis can resolve following treatment for the underlying cause of liver injury, such as long-term viral suppression in patients with hepatitis B.^2^ However, for many patients who progress to cirrhosis, a lack of response to specific therapy or the absence of effective treatment means that the only prospect for survival is liver transplantation. As the demand for liver donors continues to increase, there is an unmet need for alternatives to whole-organ transplantations.

One alternative may be human amniotic epithelial cells (hAEC) derived from the amniotic membrane which develops from the epiblast prior to gastrulation, thus retaining the capacity to differentiate into cells from all 3 germ layers. hAEC express embryonic stem cell markers but do not express telomerase, are non-tumorigenic, and do not elicit immune-mediated rejection in the host.^3,4^ We have shown that hAEC have intrinsic immune-modulating characteristics that can induce hepatic fibrosis regression in necroinflammatory liver disease models^5-7^ and steatohepatitis models.^8^ Regarding their potential for clinical use, compared to hundreds of thousands of mesenchymal stromal cells (MSC) that can be isolated from adipose tissue or bone marrow, 120–200 million hAEC are routinely isolated from a single term amnion using a method that is free of animal products and compliant with clinical use.^9^ The isolation process for hAEC is approximately 4-6 hours compared to MSC that require 4-6 weeks of ex vivo expansion to obtain a similar number of cells for therapeutic use, making hAEC a low-cost and abundant cell resource. Moreover, hAEC pose no ethical concerns as the placenta is normally discarded following delivery.

Based on our extensive pre-clinical data showing the favorable characteristics of these cells, we conducted a phase I clinical trial with the primary endpoint being the safety and tolerability of allogeneic transplantation of hAEC in adult patients with compensated cirrhosis.^10^ The secondary objectives included changes in clinical measurements of cirrhosis severity [Child-Pugh (CPT) score, Model for End-stage Liver Disease (MELD), FIB-4, liver stiffness], liver inflammation (serum AST) and portal hypertension (hepatic venous pressure gradient, HVPG) 8 weeks after hAEC administration.

## Materials and methods

### Data safety monitoring board

A data safety monitoring board (DSMB) comprising 3 independent experts, chosen for their expertise in liver disease, cell therapy, and clinical trial methodology, provided advice and recommendations to the study investigators. The primary responsibility of the DSMB was to review and evaluate the study data for participant safety, study conduct, and progress and to make recommendations to the investigators concerning the continuation, modification, or termination of the trial. The DSMB reviewed all data pertaining to each dose cohort and approved the next dose cohort.

All research was conducted in accordance with the Declaration of Helsinki. This study was approved by the Monash Health Human Research Ethics Committee (Research Project application16052A) in August 2016 and prospectively registered with the Australian New Zealand Clinical Trials Registry (ANZCTR 12616000437460). Written informed consent was obtained from all study participants and from all donor mothers who provided their placental amniotic membranes.

### Inclusion criteria

Adult female or male patients with compensated cirrhosis aged ≥18 years to ≤70 years were included in the study. Patients with metabolic dysfunction associated steatotic liver disease (MASLD), alcohol-related liver disease (abstinence for at least 3 months), hepatitis C virus infection (treated or not treated), hepatitis B virus infection (on nucleoside analogs with normal ALT and suppressed HBV DNA viral load or inactive phase), HIV co-infection with HCV/HBV with virological suppression for >12 months, cryptogenic cirrhosis, and hemochromatosis (on maintenance venesection) were able to enter the study.

### Exclusion criteria

Patients were excluded from the study if they had current or previous episodes of decompensated liver disease, including variceal hemorrhage, hepatic encephalopathy, or ascites, or if they had primary biliary cholangitis, autoimmune hepatitis, other active autoimmune disease (IgG > 2xULN), renal insufficiency (eGFR < 70 mL/minute/1.73 m^2^), HIV infection (untreated or uncontrolled viremia), HBV DNA > 200 IU/mL, fulminant hepatitis, primary sclerosing cholangitis, portal vein thrombosis, significant comorbidity (chronic heart failure, obstructive airway disease, pulmonary hypertension, or other in the investigator’s opinion), pregnancy, fibrotic liver disease other than cirrhosis, or who were unable or unwilling to provide informed consent.

Cirrhosis was defined as one of: liver biopsy confirming cirrhosis, transient elastography with a liver stiffness measurement (LSM) > 14.5 kPa, FIB 4 > 3.25, or clinical and radiological features that in the opinion of the investigator were consistent with a diagnosis of cirrhosis. Portal hypertension was assessed by measuring the hepatic venous pressure gradient with a normal portal pressure ranging from 1 to 5 mmHg.^11^

### hAEC isolation

Healthy pregnant mothers delivering at term (37-40 weeks gestation) were approached and consented to donate their amniotic membranes. The donor mothers were screened for viral and other infections on the day of delivery in accordance with Therapeutic Goods Order No. 88 guidelines. These investigations were repeated 3 months later to exclude pathogens that might have been incubating during delivery. hAECs were isolated in a GMP-compliant Xvivo isolator (Biospherix, Parish, NY, USA) housed in an ISO8 cleanroom on the cell therapy and regenerative medicine platform, Monash Health Translational Precinct. hAEC were isolated as described previously.^9^ Purity was assessed by flow cytometry using EpCam + cells (BD Biosciences). Batches that were >90% EpCam-positive but <1% positive for MSC markers (CD90 and CD105) were cryopreserved in 5% human serum albumin and 5% DMSO. The hAECs were stored in a secure and dedicated liquid nitrogen dewar until clinical release criteria were met.

### Number of cells and route of hAEC administration

We administered cells via peripheral intravenous infusion based on our experience in animal models and 6 infants treated in a separate study at Monash Health. This route appeared safe and well tolerated and was also consistent with the route of administration in published studies on MSC transplantation.^12,13^ The number of cells administered in MSC trials has generally ranged from 10^6^/kg to 10^8^/kg.^12^ We chose a low starting dose of 0.5 × 10^6^/kg body weight. Cryopreserved hAECs were thawed and washed to remove DMSO prior to formulation for infusion. The cell yield and viability of all hAEC batches used in the trial are summarized in Supplementary Table S1. All batches were screened for their ability to reduce collagen production by ≥30% in LX-2 cells (a hepatic stellate cell line) and suppress proliferation of CD3/CD28 activated T cells by ≥25%.

### Trial design

**Figure 1.**
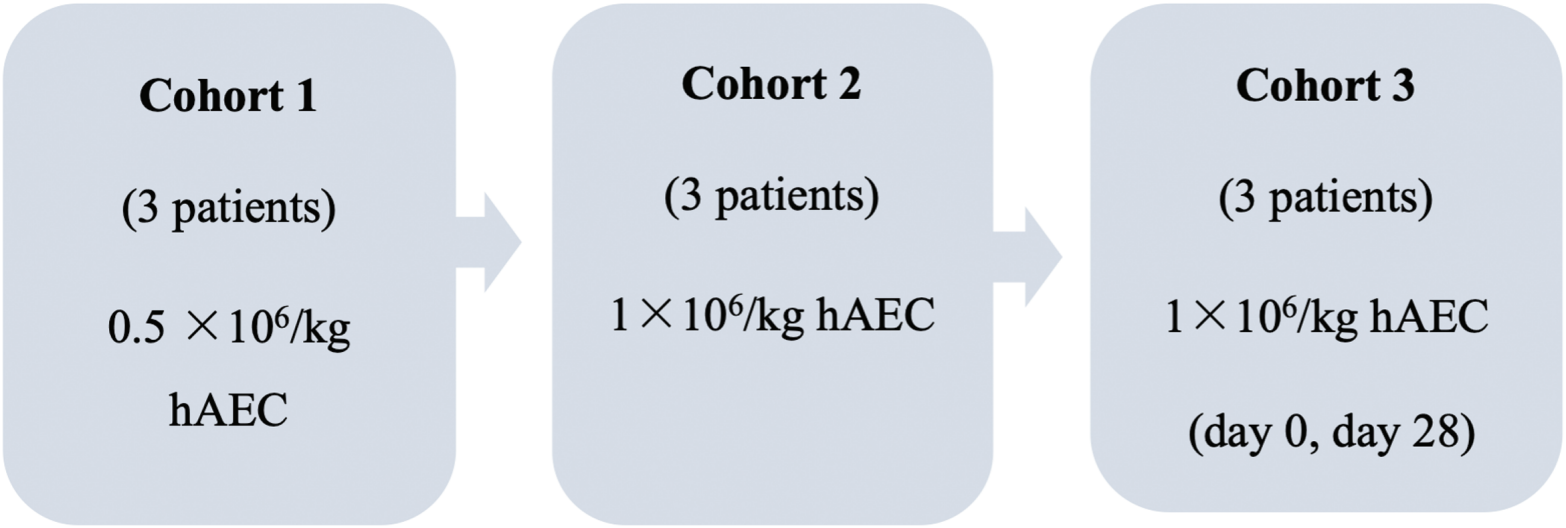
Study design. This study was designed to examine sequentially the safety of hAEC dose escalation and multiple doses. Patients in cohort 1 were administered a single infusion at the lowest hAEC dose, followed by those in cohort 2 who received double the initial dose in a single infusion. Patients in cohort 3 received 2 infusions of the higher hAEC dose, one on day 0 and the second on day 28. Progression to the next dose occurred only if no serious adverse events were observed in the 5 days following the infusion.

This study was designed to examine increasing hAEC doses as well as multiple doses over time. The patients in each cohort were treated sequentially. Each patient was then observed for serious adverse events (SAE) for 5 days post hAEC infusion before the next patient in that cohort was treated. Patients in cohort 1 received 0.5 × 10^6^/kg hAEC as a single intravenous infusion. If no SAE occurred within 5 days of infusion in the third patient, another 3 patients were sequentially enrolled to receive a higher hAEC dose of 1 × 10^6^/kg hAEC. If no SAE occurred within 5 days of infusion in the third patient in Cohort 2, a further 3 patients were sequentially enrolled to receive 1 × 10^6^/kg hAEC on days 0 and 28 (Figure 1). Cell yield and viability for each donor are described in Supplementary Table S1. Infusion volume and duration of infusion for each patient are summarized in Supplementary Tables S2-S4. Supplementary Table S5 shows each pool of donors used, the pooled pre- and post-infusion cell number and viability for each infusion and each patient enrolled in the trial.

### Assessment of portal venous pressure using the hepatic venous pressure gradient

To measure the pressure gradient between the hepatic vein and portal vein, a balloon-tipped catheter was introduced into the hepatic vein, usually by a right internal jugular vein approach, and free pressure was measured. The balloon was inflated and “wedged” in the hepatic vein, and the measured pressure directly reflected the hepatic sinusoidal pressure and thus portal vein pressure. The difference between the wedged and free hepatic vein pressures represents the HVPG. Values less than 6 mmHg were considered normal, values ≥ 6 indicated portal hypertension, and values ≥ 10 indicated clinically significant portal hypertension.^11,14^

### Non-invasive testing

Non-invasive estimation of liver fibrosis was determined by liver stiffness measurement (LSM) using transient elastography (FibroScan Echosens, Paris). LSM ≥ 14.5 kPa was used as the cutoff in this study for the diagnosis of cirrhosis. Any potential patient with values of < 14.5 kPa required another method to confirm cirrhosis. Serum constituents related to fibrogenesis (extracellular matrix components), liver function (prothrombin time or the international normalized ratio), and portal hypertension (platelets) have been used to assess liver fibrosis in common conditions, such as viral hepatitis and MASLD. The FIB-4 test includes age, serum AST and ALT levels, and platelet count. Values > 3.25 have good accuracy in determining cirrhosis in HCV infection and alcoholic liver disease. Any potential patient with values below 3.25 required an additional method to confirm cirrhosis.

### Statistical analysis

No formal statistical analysis of this phase I trial has been conducted. Descriptive statistics were used to summarize the features of the dataset.

### Patient monitoring

Patients were observed during and following cell infusion at the Clinical Trial Center of the Monash Health Translational Research Facility, with appropriate monitoring facilities for 24 hours. Blood pressure and pulse oximetry monitoring were performed every 15 minutes during the first 2 hours, every 30 minutes for the next 2 hours, and then hourly for 24 hours. Body temperature was measured every 30 minutes for 4 hours and hourly for 24 hours.

Daily telephone contact to assess the overall status occurred on post-infusion days 2–4. On day 5 post-infusion, patients returned to the Clinical Trial Center for assessment, including open-ended questions to ascertain any adverse events, record concomitant medications, and obtain blood for laboratory measurement of hepatic, hematologic, and renal function. A 10 mL serum sample was retained for storage.

During post-infusion week 8 for cohorts 1 and 2, and week 12 for cohort 3, routine laboratory tests were performed on day 5. FibroScan and HVPG measurements were repeated and a serum sample (10 mL) was obtained for storage.

### Safety assessment

Safety analyses were summarized according to the cohort and treatment groups. Standard physical examinations, vital signs, electrocardiograms, and clinical laboratory tests were performed during study visits. Suspected unexpected serious adverse event reporting (SUSAR) was utilized. Adverse events (AEs) were recorded from day 0 to the end of follow-up and classified by severity as grades 1, 2, 3, or 4 using the National Cancer Institute Common Terminology Criteria for Adverse Events (v5.0). The incidence and frequency of AEs and serious AEs and their relationship to the study drug, including those leading to patient withdrawal, dose modification, or treatment discontinuation, were categorized by dose and treatment group. Treatment-emergent AEs were summarized using the latest version of the medical dictionary for regulatory activities, and their severity was classified as mild, moderate, or severe.

### Events of special interest

In addition to the standard adverse event determination, we were interested in specific events, including but not limited to (1) anaphylaxis, acute deterioration of respiratory, or cardiovascular parameters; (2) respiratory deterioration, dyspnea, hypoxemia, or any requirement for supplemental oxygen, or mechanical ventilation; (3) cardiovascular deterioration, hypotension, hypertension, bradycardia, tachycardia, rhythm abnormalities, or (4) worsening organ function as shown by impairment of hepatic or renal parameters.

## Results

### Study population

Fifty patients were invited to participate in the study between December 2016 and December 2021. Of these, 21 patients declined and 15 met the exclusion criteria. Of the remaining 14 patients, 5 failed screening, including 3 patients with HVPG > 12 mmHg (patients 004, 005, 013) and 1 (008) who withdrew consent. One patient (003) initially failed screening due to an elevated ESR; however, following subsequent observations was rescreened and commenced in cohort 2. Nine patients who met all inclusion criteria and no exclusion criteria were included in the study. We originally planned a fourth cohort of patients to receive 3 hAEC doses (days 0, 28, and 56), but this was not achieved owing to COVID restrictions on study recruitment at our trial center.

### Baseline demographics

Four women and 5 men completed all study procedures. The median age was 57 years (range, 38-67 years), and 8 patients had MASLD as a cause of their liver disease (Table 1). One patient (001) had previous successful eradication of hepatitis C infection. All patients had compensated liver disease based on Child-Pugh and MELD scores. Hepatic fibrosis was assessed using invasive and non-invasive measurements. Four patients had previously undergone a liver biopsy. Non-invasive testing for fibrosis included the FIB-4 test and measurement of liver stiffness using transient elastography. All patients had their HVPG measured at baseline (Table 2).

**Table 1.** Baseline demographics.

Patient number	Sex	Age (years)	Body mass index	Liver disease etiology	CPT^c^	MELD^d^
001	M	38	21	HCV^a^	A5	7
002	M	63	23	MASLD^b^	A5	9
006	M	57	36	MASLD	A5	10
007	F	59	32	MASLD	A5	8
003	F	52	44	MASLD	A5	6
009	M	56	37	MASLD	A5	8
010	M	57	43	MASLD	A6	7
012	F	47	43	MASLD	A5	7
014	F	67	28	MASLD	A5	6

^a^Hepatitis C virus.

^b^Metabolic-associated steatotic liver disease.

^c^Child Pugh Turcotte score.

^d^Model for end stage liver disease.

**Table 2.** Hepatic fibrosis and portal hypertension measurements at baseline.

Patient number	Cohort/	Liver histology stage	FIB4^a^	LSM^b^	HVPG
	Patient number			(kPa)	(mmHg)
001	C1/Pt 1	F4	1.57	6.7	6
002	C1/Pt 2	—	3.38	16.6	10
006	C1/Pt 3	—	3.5	30.9	10
007	C2/Pt 1	—	2.18	16.8	8
003	C2/Pt 2	—	0.98	28.6	8
009	C2/Pt 3	F4	2.91	75	5
010	C3/Pt 1	F4	3.34	33.2	11
012	C3/Pt 2	—	1.97	22.8	6
014	C3/Pt 3	F4	1.79	12.4	7

^a^FIB-4 score.

^b^Liver stiffness measurement (kiloPascal).

^c^Hepatic venous pressure gradient (millimetres of mercury).

### Safety and tolerability

No serious adverse events occurred during the study period in any of the patients. Six patients experienced no study-related adverse events (patients 002, 006, 009, 010, 012, and 014). Three patients reported headaches that were possibly infusion-related. The headaches were mild (grade 1) and occurred at variable times following cell infusion (patient 001 at 255 minutes, patient 003 at 54 minutes, and patient 007 at 6 hours). Blurred vision occurred in patient 001, who was infused with 34.6 million cells over 25 minutes. At 5 minutes post-dose, he experienced a 5-minute episode of lightheadedness. At 78 minutes post-dose, he experienced intermittent dizziness, sweating, blurred vision, and loss of visual focus for 11 minutes, which resolved spontaneously. He was seen within 2 hours by the ophthalmology service, who found his ocular examination, including visual acuity, to be within normal limits. When the patient was again reviewed by the ophthalmology service 9 days later, his ocular examination results remained within normal limits. These symptoms were assessed as possibly related given their proximity to cell infusion. Considering that these symptoms might be related to cell clumping in the microvasculature subsequent infusions included an in-line filter inserted in the giving set and the bag of cells was placed on a rocking platform rather than suspended on an intravenous pole. Other events judged as possibly or probably related were reported in a single patient and included transient loose bowel actions (grade 1) and muscle aches (grade 1) in patient 007. Two patients reported heightened energy levels and decreased lethargy on the days following infusion. All infusions were performed in less than 4 hours, a length of time that formulated hAECs have been found to maintain their viability at room temperature (unpublished data).

We observed a reversible decrease in serum platelet levels in all patients during the study period. Interestingly, a decrease was observed prior to cell infusion when screening values were compared with the pre-infusion values obtained on the day of infusion (median decrease, 6.5%; range, 2%-19%). There was a greater decrease in platelet count when the same pre-infusion values were compared with those at 30 minutes post-infusion on the day of infusion (median decrease, 14.9%; range, 3.5%-32.9%). Platelet levels returned to near or above the baseline screening values by day 5 post-infusion. No clinical events were associated with the transient changes in platelet levels. Serum aminotransferase (AST, ALT) levels did not increase >2-fold from baseline in any patient. Similarly, there were no reported increases in total bilirubin other than in 3 patients with baseline elevations of total and indirect bilirubin; direct-acting bilirubin values were normal in these 3 patients, suggesting underlying Gilbert syndrome.

### Non-invasive markers of hepatic inflammation and fibrosis

**Figure 2.**
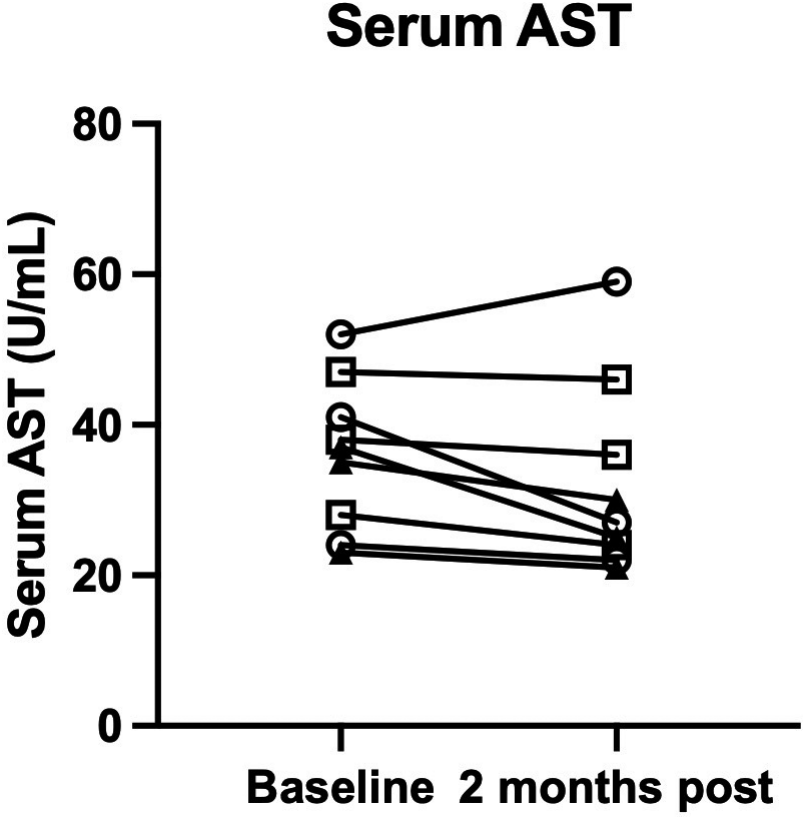
Serum AST at day 58. Median serum AST values were either lower or stable in 8 patients at day 58 compared with baseline values. One patient showed an AST increase from 52 to 58 U/L. Data points from the first cohort are represented by an open circle, data points from the second cohort are represented by a closed triangle, and data points from the third cohort are represented by an open square.

**Figure 3.**
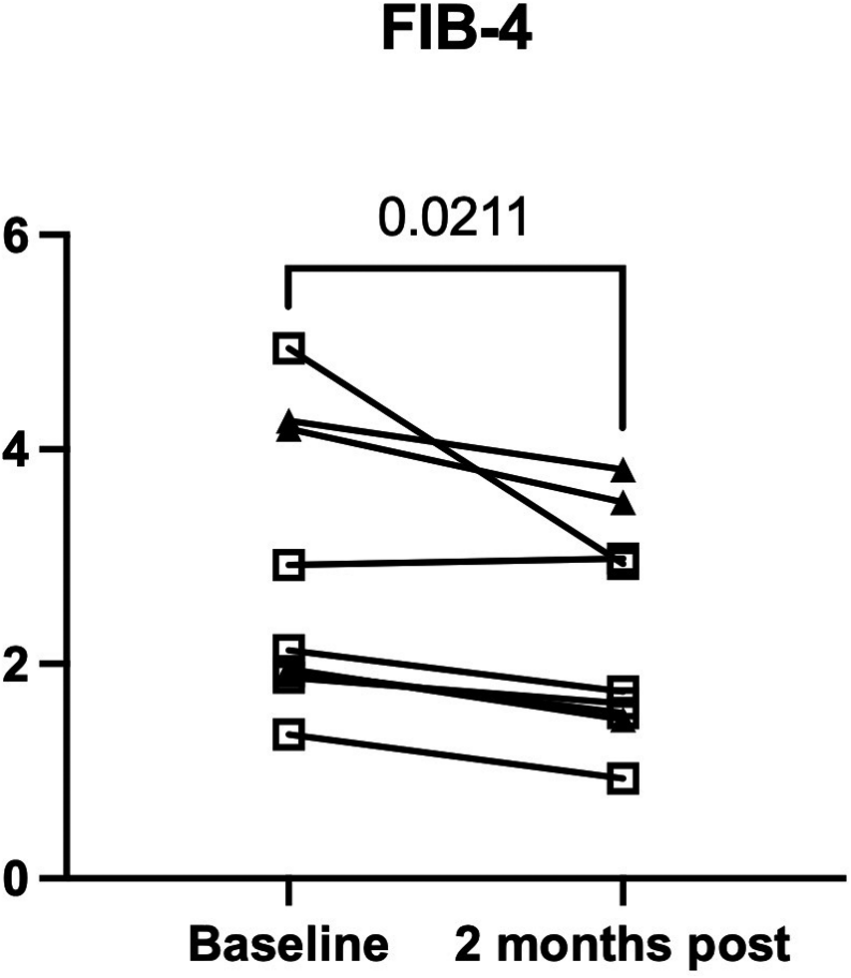
Fib-4 scores at day 58. FIB-4 values were lower at day 58 following hAEC infusion compared with baseline values (**P = .02). Data points from the first cohort are represented by an open circle, the second cohort are represented by a closed triangle, and data points from the third cohort are represented by an open square.

We examined changes in commonly used serum markers, such as serum AST and ALT, associated with hepatic inflammation and fibrosis. These enzymes are incorporated into many non-invasive fibrosis algorithms, such as the AST to platelet ratio (APRI), FIB-4, and the FibroScan-AST (FAST) score. The reference range for AST in our hospital laboratory is 3-35 U/L. AST elevation greater than 2-fold (70 U/L) was not detected during the study period; rather, we observed either stable or decreased AST values between baseline and 2 months after cell infusion in 8 patients, while 1 patient increased from 52 to 58 U/L (Figure 2). In comparison, the values for serum ALT (reference range, 5-40 U/L) were similar at baseline and 2 months post-infusion (data not shown). FIB-4 is a non-invasive assessment of hepatic fibrosis, which includes age, serum ALT, AST, and platelets. We identified a significant decrease in baseline FIB-4 values compared to those at 2 months following hAEC infusion (Figure 3). In Figure 3, symbols were allocated to data points from each cohort. Data points from the first cohort are represented by an open circle (❍), those for the second cohort are represented by a closed triangle (▲), and data points for the third cohort are represented by an open square (□).

### Liver stiffness measurement

**Figure 4.**
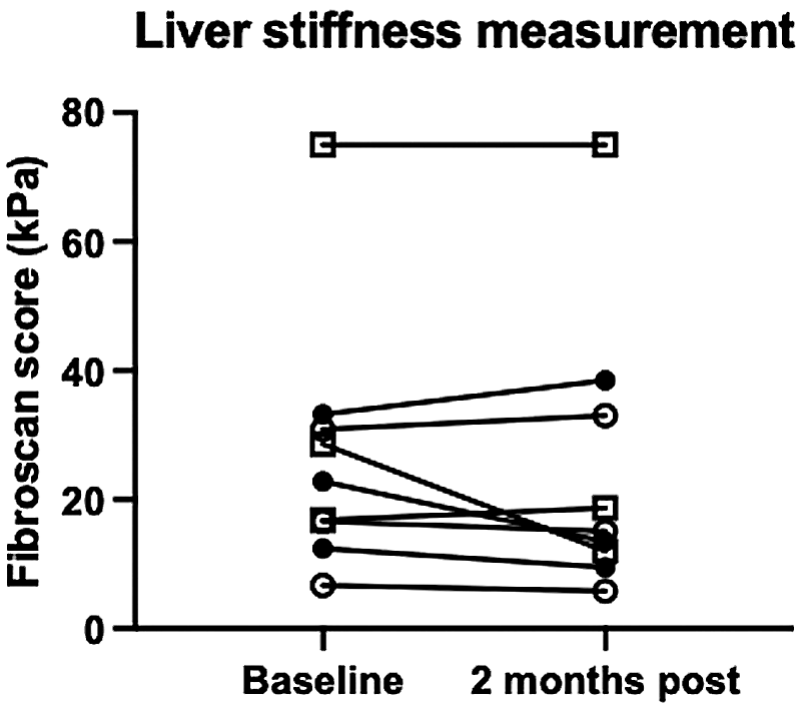
Liver stiffness measurements at day 58. At 2 months following hAEC infusion, liver stiffness measurements were lower in 6 patients, increased in 2 patients and elevated but stable in a third patient. Data points from the first cohort are represented by an open circle, data points from the second cohort are represented by a closed triangle, and data points for the third cohort are represented by an open square.

Liver stiffness was measured by transient elastography during screening, 5 days following hAEC infusion, and again 2 months following infusion. While the values were lower in 6 patients 2 months after infusion, the overall differences were not significant. One patient (009) with a high baseline LSM of 75 kPa showed no change following hAEC exposure (Figure 4).

### Hepatic venous pressure gradient

**Figure 5.**
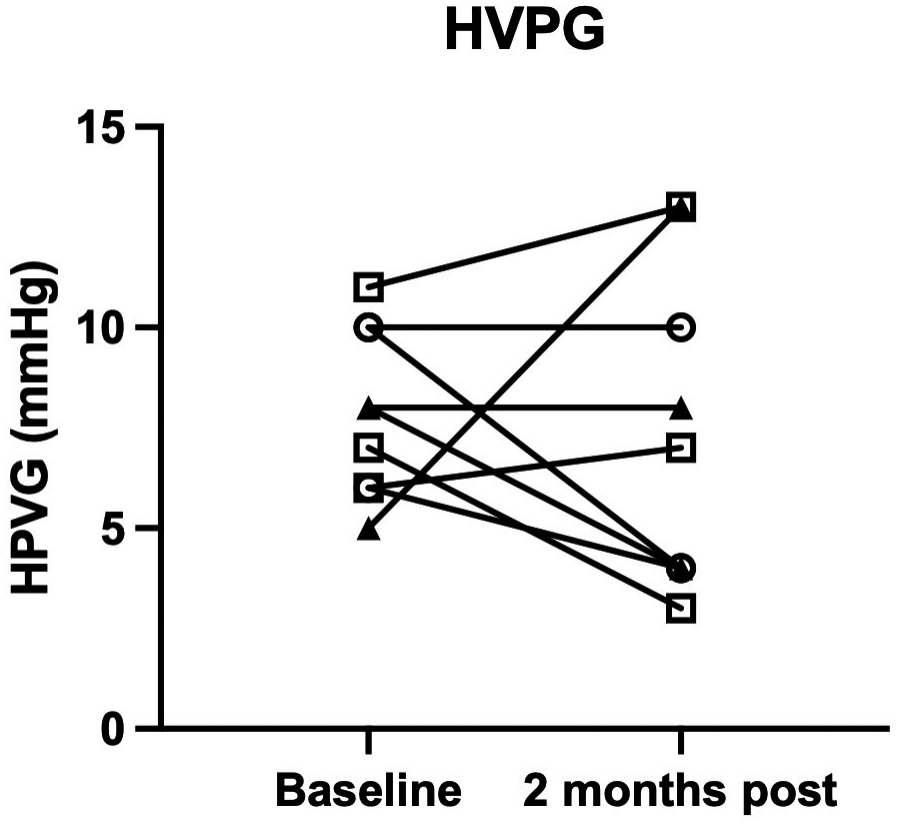
Hepatic venous pressure gradients at day 58. The hepatic portal venous pressure gradient (HVPG) decreased in 4 patients at 2 months post-infusion and showed either no change or a small increase in 4 patients. One patient demonstrated a large increase in HVPG (see text for discussion). Data points from the first cohort are represented by an open circle, data points from the second cohort are represented by a closed triangle, and data points from the third cohort are represented by an open square.

Portal venous hypertension is a major driver of liver disease progression to decompensation and hepatocellular carcinoma. We measured portal vein pressure in each patient during screening and 2 months following hAEC infusion by determining the hepatic venous pressure gradient, which is the difference between the wedged hepatic vein pressure (an indirect measurement of portal vein pressure) corrected by an internal zero (free hepatic vein pressure).^11^ The normal gradient is 3-5 mmHg, whereas a value ≥10 mmHg is defined as clinically significant portal hypertension.^14^ We found that the HVPG was elevated at baseline in all patients, except for patient 009, who had a normal pressure at baseline (5 mmHg). This normal value is likely due to a technical variance in measurement, as this patient was clinically cirrhotic with portal hypertension based on ultrasound findings, including splenomegaly, LSM of 75 kPa, and low serum platelets on repeated measurements. At 2 months post-infusion, patient 009 showed an increase in the HVPG from 5 to 13 mmHg, with the latter value likely reflecting the true portal pressure gradient. In 4 patients, there was a decline in the HVPG at 2 months. Four patients showed either no change or a small increase in HVPG (Figure 5).

### Model for end-stage liver disease

**Figure 6.**
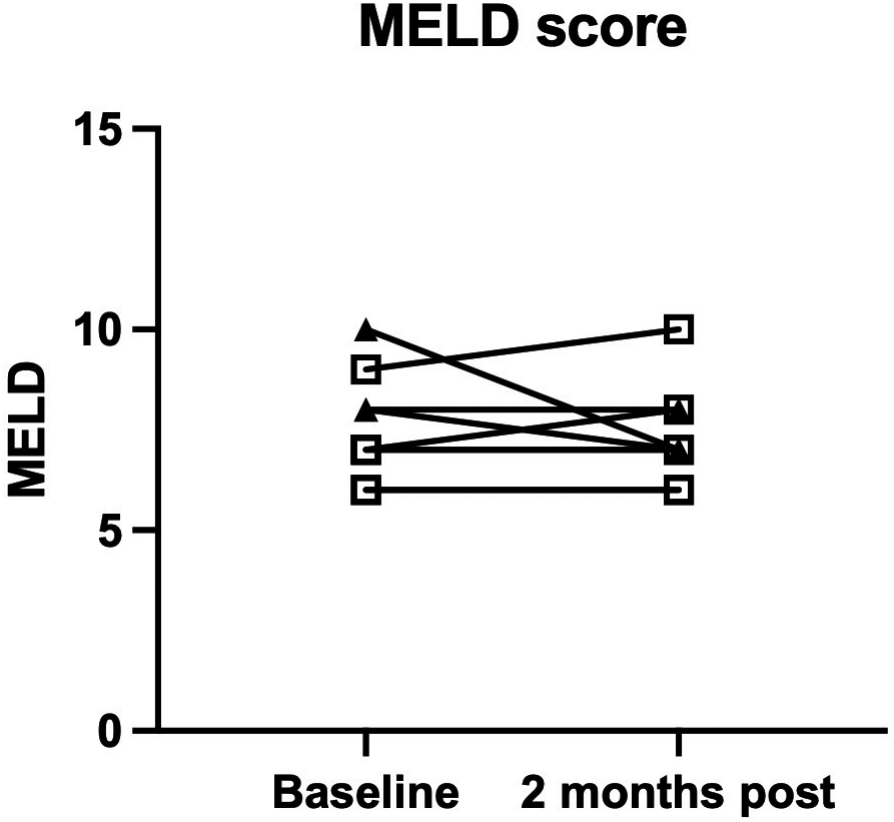
Model for end-stage liver disease at day 58. MELD was unchanged in 5 patients at 2 months post-infusion and showed either no change or a small increase in 4 patients. Data points from the first cohort are represented by an open circle, data points from the second cohort are represented by a closed triangle, and data points from the third cohort are represented by an open square.

Between baseline and 2 months post-infusion, the MELD score was unchanged in 5 patients, increased by 1 point in 2 patients, and decreased in 2 patients by 1 and 3 points, respectively. (Figure 6).

## Discussion

In this phase I study, the first in patients with cirrhosis, we showed that intravenous infusion of allogeneic hAEC, up to 10^6^ cells/kg in 2 separate doses, was safe and well-tolerated. Six of the 9 patients experienced no adverse clinical events. Three patients experienced grade 1 headaches; one of these patients also experienced transient blurred vision, and one reported transient loose bowel actions and muscle aches, both of which were grade 1. Two patients reported heightened energy levels and decreased lethargy on the days following infusion.

In the first cohort, 3 patients were dosed by weight at 0.5 × 10^6^ cells/kg, modeled on a commonly used dosing strategy in clinical trials based on mesenchymal stromal cells (MSC) which are frequently injected in doses ranging from 1 × 10^6^ to 1 × 10^8^ cells/kg. The dose was then escalated to 1 × 10^6^ cells/kg in the second cohort of 3 patients. In the third cohort, 2 doses of 1 × 10^6^ cells/kg were administered. No safety signals were observed in patients who received the highest dose. We observed a transient decrease in serum platelet levels in all patients, noting that the initial decline occurred during the screening period prior to hAEC exposure. When comparing the screening values with the pre-infusion values obtained on the day of infusion, a median decrease of 6.5% (range, 2%-19%) was observed. When the same pre-infusion values were compared with those at 30 minutes post-infusion, the median decrease was 14.9% (range 3.5%-32.9%). All platelet counts returned to near or above the baseline screening values by day 5 post infusion. While the cause of these changes remains unclear, a similar decrease in platelets in a cohort of cirrhotic HBV-infected patients administered umbilical cord MSC was recently reported.^15^ Importantly, the observed decreases were reversible and no clinical events occurred in relation to changes in platelet levels.

As an exploratory outcome, we were interested in whether changes occurred in hepatic inflammation or fibrosis measurements after hAEC infusion. Serum aminotransferase levels were compared between baseline and day 58. While there was no difference in the serum ALT values, the serum AST values were lower on day 58 than at baseline (Figure 2). We observed significantly lower FIB-4 values 2 months after hAEC infusion (Figure 3). Similarly, liver stiffness was lower on day 58 than the baseline values (Figure 4). Comparing individual HVPG outcomes at day 58, the values were lower in 4 patients, did not change in 2 patients, and increased in 3 patients (Figure 5). MELD values were lower on day 58 than those at baseline (Figure 6). While it is not possible to reach definitive conclusions regarding a beneficial therapeutic effect of hAEC, or lack of one, from this small patient cohort, there is a consistent trend that, given the safety data, supports further studies of hAEC-based treatment in patients with compensated and decompensated cirrhosis.

It is worth noting that, in a variation from the original study protocol,^10^ the fourth cohort of 3 patients planned to receive 3 doses of 1 × 10^6^ cells/kg was not recruited due to significant prolongation of the study duration based on COVID-19 restrictions limiting patient admissions into our clinical trial center. From March 2020 to October 2021, Melbourne, Victoria, was subjected to 6 periods of lockdown to reduce COVID-19 infections. During this period, significant limitations were placed on non-urgent hospital admissions, including clinical trials; thus, the trial was terminated without enrolment of a fourth cohort.

Acknowledging the extensive experience with mesenchymal stromal cell transplantation in cirrhosis, is another type of cell-based therapy required? The safety and efficacy of MSCs have been evaluated in multiple clinical trials. A recent systematic review and meta-analysis of randomized controlled trials by Liu et al showed that MSC therapy improved liver function (assessed by MELD and other measures) compared with standard therapy, albeit with substantial data heterogeneity among studies.^16^ The review found that while individual studies did not show an improvement in survival, there was a statistically significant effect on survival when the studies were combined (pooled OR 1.29, *P* = .023). Similar outcomes were identified for functional measures such as serum albumin, total bilirubin, and coagulation factors. Importantly, MSC were found to be safe with no serious adverse events or related deaths identified; fever was the only reported side effect in 5 of the 12 included studies. Although the authors concluded that MSCs are considered the most promising cells for liver regeneration, transplantation, and cell therapy, there are potential disadvantages to their large-scale use. For example, some studies have suggested that MSC can play a role in promoting a variety of tumors, including lung, breast, and myeloma,^17^ and may differentiate into myofibroblasts that promote fibrogenesis.^18^ At a practical level, MSC acquisition is often invasive and may result in procedure-related complications.^19^ The relative numbers harvested may be small, necessitating extensive ex vivo expansion, which may lead to genetic changes that predispose patients to carcinogenesis and thus require a risk-mitigation strategy such as costly in-processing checks for genetic and karyotypic stability. Serial passaging can also result in MSC senescence, which may hinder their clinical application. Finally, the injected cell population may contain many different cell types in addition to MSC, such as endothelial progenitors, T and B lymphocytes, and natural killer cells,^20^ whereas we used a well-characterized population of >90% pure hAEC (Supplementary Figure).

In addition to MSC, other cell types have been considered as potential therapeutic agents in patients with chronic liver disease including induced pluripotent stem cells, hepatic progenitor cells, and allogeneic hepatocytes. Strom et al^21^ reported on the efficacy of hepatocyte transplantation in patients with fulminant hepatic failure and acute decompensation of chronic liver failure. While the number of reported patients was small (*n* = 20), there was an overall survival advantage for those receiving transplanted hepatocytes. Other authors have suggested that hepatocyte transplantation might be optimized through co-transplantation with MSCs or other cell types and the concomitant use of cytokines to enhance cell function.^22,23^ Challenges include a reliable supply of hepatocytes and, while full recovery has been observed, there is a lack of clinical trials to support efficacy.^24^

The route of cell administration varies among MSC studies, with peripheral vein injection being a commonly used method. Other routes, such as hepatic arterial injection, have been postulated to be more effective than peripheral vein injection, owing to a more targeted distribution of the cellular product. However, this is an invasive approach, with risks of portal hypertensive bleeding and thrombosis. To date, there is no consensus regarding the optimal route of cell administration^25^ so we chose peripheral vein injection as a less complex and more patient acceptable process for this early-phase safety study. Additionally, there is no consensus on the potential benefits of repeated MSC dosing. A meta-analysis^15^ concluded that multiple MSC administration showed a greater benefit on MELD scores, while a single MSC administration resulted in improved serum albumin levels. Furthermore, the replacement of hepatocytes by acellular extracellular matrix in cirrhotic livers may pose a physical barrier to the therapeutic biodistribution of peripherally administered cells. However, we have shown in an immunocompetent (C57Bl6) murine hepatic fibrosis model induced by carbon tetrachloride that hAEC injected via the tail vein were identified in the liver. These cells remained viable 2 weeks following infusion with human albumin detected in murine sera from all hAEC-treated animals,^5^ suggesting successful xenotransplantation, engraftment, and differentiation in the absence of immunosuppressants.

We hypothesized that hAEC would be beneficial in treating patients with cirrhosis as an alternative to either whole organ transplantation or MSC transplantation. The fetal amniotic membrane from which hAEC are derived has been used for wound healing since early in the 20th century.^26^ In 1940 an ophthalmic surgeon reported their use in repair of conjunctival defects and noted, “the embryonal tissue used has a property of being transformed to conjunctiva.”^27^ Amniotic epithelial cells express embryonic stem cell markers, but do not express telomerase, are non-tumorigenic, and have multi-lineage differentiation potential into cells derived from the 3 germ layers,^3,4^ thus demonstrating pluripotent capacity. We extensively characterized the effects of hAEC in cell-based animal models of fibrosis and cirrhosis. In our in vivo studies, hAEC were administered to animals with established cirrhosis and continuing liver injury to model the clinical scenario of patients presenting with cirrhosis and ongoing liver injury. We showed that hAEC have potent anti-inflammatory and anti-fibrotic properties in these rigorous animal models.^5-8^ Unlike potential anti-fibrotic agents that target a single-cell type or pathway, hAEC appear to decrease inflammation and fibrosis by interacting with several cell types, including hepatic stellate cells,^5,7^ macrophages,^6^ neutrophils^28^, and liver progenitor cells.^29,30^ This raises the question of whether hAEC achieve a beneficial trophic effect by differentiating into hepatocytes that proliferate to replace the diminished hepatic parenchyma in cirrhotic patients, or primarily exert anti-inflammatory and anti-fibrotic effects that promote recovery through activation of endogenous liver progenitor cells that expand and differentiate to increase the resident hepatocyte population. Both mechanisms may restore liver function.

Clinical applications of cell-based therapies for liver diseases have recently been reviewed. Several cell sources exist, including hepatocytes isolated from donor livers that are not suitable for transplantation, MSC, hepatic progenitor cells, and induced pluripotent stem cells.^16,19^ Challenges for these cell types include the length of time required for ex vivo manipulation to achieve sufficient numbers of viable cells, scalability to meet commercial demands, and standardization of these procedures. As previously mentioned, the route of administration varies from complex, including hepatic artery injection, portal vein infusion, or splenic injection, to the simple use of peripheral intravenous administration. While we used peripheral vein infusion in this study, more liver-directed administration via the spleen, portal vein, or the hepatic artery may result in greater liver engraftment and thus greater efficacy. We will explore this in future studies. An emerging area of interest is the delivery of biologically active cell products, such as extracellular vesicles (EV). In animal models, we have shown that hAEC-conditioned media and EV derived from hAEC replicate many of the experimental outcomes observed with whole-cell infusion, including reduction of inflammation and fibrosis.^28,29,31^ EV-based therapies can avoid some of the challenges with cell-based therapy mentioned above, with fewer logistical barriers at the clinical level, through the formulation of lyophilized products.

Regulatory approval for these new therapeutics will be based on trials powered to detect clinically meaningful improvements in trial endpoints that validate such benefits. It is important to note that the trial results must be sufficient to demonstrate the magnitude and duration of the response. We are planning phase II studies that will involve a larger number of participants to determine relevant clinical efficacy. A major requirement is evidence that the product has a demonstrable impact on a surrogate endpoint or intermediate clinical endpoint that is reasonably predictive of clinical benefit and superior to available therapy. In the case of cirrhosis, it would be reasonable for later-phase efficacy trials to include well-characterized indicators such as MELD, FIB-4, and HVPG as surrogate endpoints and ultimately show a reduction in clinical decompensation episodes and greater transplant-free survival as indicators of overall clinical benefit.

## Conclusions

Peripheral intravenous infusion of allogeneic hAEC in patients with compensated cirrhosis at the doses used in this study was safe and well tolerated. Similar reassuring outcomes were also observed in a recent study from our center in 8 patients with acute stroke who received hAEC.^32^ We did not identify a clear difference in outcomes between patients who received a single dose and those who received 2 doses. Although no formal statistical analysis was planned for this small group of patients, there were promising signals of decreased hepatic inflammation, liver stiffness, and portal hypertension that would support larger studies in patients with compensated and decompensated cirrhosis to understand which patients are likely to benefit, as well as the optimal dose and method to deliver an effective treatment for hepatic fibrosis.

## Supplementary Material

Supplementary material is available at *Stem Cells Translational Medicine* online.

szae023_suppl_Supplementary_Tables_1-5

## Data Availability

The data underlying this article will be shared on reasonable request to the corresponding author.
