# Supplementary material for: Human Amniotic Epithelial Cell Transplantation is Safe and Well Tolerated in Patients with Compensated Cirrhosis: A First-in-Human Trial
Source: Stem Cells Transl Med. 2024 Jun 10;13(6):522–31. doi: 10.1093/stcltm/szae023 (PMC11165158; doi:10.1093/stcltm/szae023)
Supplement: szae023_suppl_Supplementary_Tables_1-5 [file szae023_suppl_supplementary_tables_1-5.pdf]

Supplementary Figures and Tables

Supplementary Figure

Representative flow cytometry characteristics of hAECs used. Single cells were selected using gates against forward vs side scatter (A) followed by forward area vs height (B). A live/dead dye was used to identify viable cells using dye exclusion (C). hAECs typically presented with <1% CD105+ (D), <1% CD34+ (E), <3% CD45+ (F) and >80% CK7+ (G), >95% HLA-G+ (H).

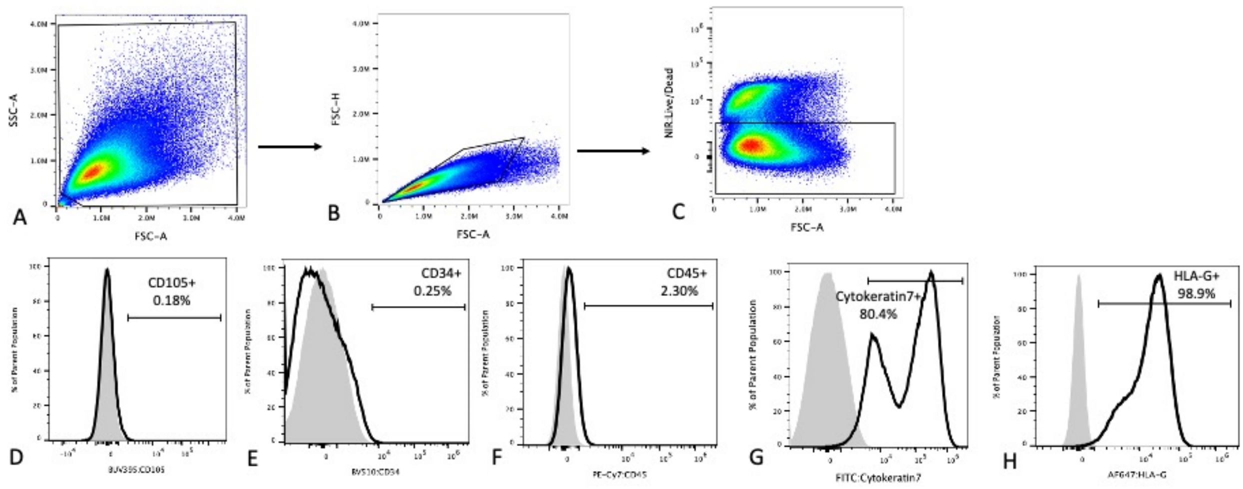

Supplementary Table 1. Cell yield and viability for each hAEC donor batch used in the study.

Donor ID	Cell Yield (x10^6)	Cell Viability (%)
2017hAEC13	178.5	75.7
2017hAEC22	55.0	83.6
2018hAEC24	86.5	92.4
2018hAEC31	113.8	64.0
2018hAEC33	223.0	92.0
2018hAEC35	98.0	84.8
2018hAEC36	258.5	76.4
2018hAEC38	117.2	72.6
2018hAEC43	138.8	85.2
2018hAEC48	131.2	92.0
2019hAEC51	111.8	86.3
2019hAEC67	120.5	82.8
2019hAEC77	124.5	78.3
2020hAEC126	264.8	79.0
2020hAEC164	147.3	84.4
2020hAEC184	468.2	82.2
2020hAEC202	145.2	84.4
2020hAEC209	217.5	86.0
2020hAEC210	164.0	89.9
2021hAEC251	156.0	86.5

Supplementary Table 2. Infusion record for Cohort 1

Infusion record Cohort 1 (single dose, 0.5x 10^6/kg)			
Patient	001-001 (C1P1)	001-002 (C1P2)	001-006 (C1P3)
Duration of infusion (minutes)	25	33	49
Target infused (millions of cells)	36	35.2	57.4
Target volume infused (mL)	144	140	229.4
Actual cell infusion (millions of cells)	33.46	43.75	67.87
Actual infusion volume (mL)	117	140	Not recorded

Supplementary Table 3. Infusion record for Cohort 2

Infusion record Cohort 2 (single dose, 1.0x10^6/kg)			
Patient	001-007 (C2P1)	001-003 (C2P2)	001-009 (C2P3)
Duration of infusion (minutes)	84	91	89
Target infused (millions of cells)	94	132.7	108
Target volume infused (mL)	388	438	432
Actual cell infusion (millions of cells)	115.6	132.7	145
Actual infusion volume (mL)	378	438	457

Supplementary Table 4. Infusion record for Cohort 3

Infusion record Cohort 3 (two doses, 1.0x10^6/kg) **Infusion 1**			
Patient	001-010	001-012	001-014
Duration of infusion (minutes)	113	100	53
Target infused (millions of cells)	134.5	120.5	63.5
Target volume infused (mL)	538	482	254
Actual cell infusion (millions of cells)	166.53	155.5	79.6
Actual infusion volume (mL)	534.9	102	272

**Infusion 2**			
Patient	001-010	001-012	001-014
Duration of infusion (minutes)	114	102	53
Target infused (millions of cells)	135.8	120.5	64.1
Target volume infused (mL)	543.2	482	256.4
Actual cell infusion (millions of cells)	170.2	130.9	83
Actual infusion volume (mL)	545.5	47604	272

Supplementary Table 5. Each donor pool used for each patient and the pooled pre and post-infusion cell numbers and viability for each infusion and each patient

Patient number	Donor ID	Cell Yield (x10^6)		Cell Viability (%)	
		Pre-infusion	Post-infusion	Pre-infusion	Post-infusion
001	2017hAEC22<br>2018hAEC33<br>2018hAEC36	67.2	10.6	79.4	80.0
002	2018hAEC33<br>2018hAEC35<br>2018hAEC36	51.8	6.9	71.9	73.5
006	2018hAEC33<br>2018hAEC35<br>2018hAEC36	85.7	17.8	74.5	62.0
007	2018hAEC33<br>2018hAEC35<br>2018hAEC36	130.0	14.4	72.5	68.0
003	2018hAEC31<br>2018hAEC33<br>2018hAEC36	137.0	Data not collected*	67.2	Data not collected*
009	2018hAEC31<br>2018hAEC38<br>2018hAEC48	145.0	Data not collected*	66.0	Data not collected*
010, cycle 1	2017hAEC13<br>2018hAEC24<br>2019hAEC51<br>2019hAEC67	168.0	1.47	73.1	62.5
010, cycle 2	2017hAEC13<br>2018hAEC43<br>2019hAEC77	171.0	0.8	73.7	68.3
012, cycle 1	2020hAEC164<br>2020hAEC184<br>2020hAEC210	158.5	3.0	70.9	63.8
012, cycle 2	2020hAEC126<br>2020hAEC184<br>2020hAEC210	162.0	31.1	76.9	64.9
014, cycle 1	2020hAEC202<br>2020hAEC209<br>2021hAEC251	119.8	40.2	74.4	69.1
014, cycle 2	2020hAEC202<br>2020hAEC209<br>2021hAEC251	137.3	54.3	76.5	58.0
